# *Orientia tsutsugamushi*: A neglected but fascinating obligate intracellular bacterial pathogen

**DOI:** 10.1371/journal.ppat.1006657

**Published:** 2017-12-07

**Authors:** Jeanne Salje

**Affiliations:** 1 Centre for Tropical Medicine and Global Health, Nuffield Department of Medicine, University of Oxford, Oxford, United Kingdom; 2 Mahidol-Oxford Tropical Medicine Research Unit, Faculty of Tropical Medicine, Mahidol University, Bangkok, Thailand; Nanyang Technological University, SINGAPORE

## Introduction

Our understanding of the molecular systems that bacteria have developed over millennia of evolutionary tinkering remains limited compared with the incredible diversity of the bacterial kingdom. A detailed understanding of the tricks and tools developed by the bacterial world provides insights into fundamental processes in biology (e.g., transcriptional networks), provides researchers with inspiration and molecular reagents for synthetic biology (e.g., restriction enzymes and CRISPR/Cas systems), and arms us with an understanding of essential bacterial processes that can be undermined by antibiotic therapy. In the case of bacteria that coexist intimately with mammalian hosts, in particular within the intracellular niche, a detailed study of these organisms can yield insights into important processes in eukaryotic biology (e.g., actin polymerisation and autophagy) [1]. However, whilst a number of model organisms have been studied in enormous detail, there remain a wealth of pathogenic and nonpathogenic bacteria whose molecular secrets remain largely unknown. This is partly due to our focus on species that inflict a high burden of human and agricultural damage and partly due to the difficulty of dissecting the molecular mechanisms of nonmodel bacterial species, which often cannot be propagated easily under laboratory conditions or manipulated genetically.

The *Rickettsia*-related bacterium *Orientia tsutsugamushi* is an example of an important human pathogen whose fundamental cell biology is poorly understood compared with other pathogens of equivalent prevalence and severity. Research is hampered by a lack of availability of tools for genetic manipulation, technical limitations associated with working with an obligate intracellular bacterium, and the cost and logistical challenges of working with a bacterium classified as a biosafety level 3 pathogen. However, there are multiple aspects of the biology of this organism that are unusual and intriguing and that can be used to address fundamental questions in host–pathogen and bacterial cell biology, and this serves to illustrate the value of taking up the challenge to study nonclassical model systems. In this review, I have highlighted some particularly fascinating aspects of the biology of this neglected intracellular pathogen.

### *O*. *tsutsugamushi* and scrub typhus

*O*. *tsutsugamushi* is a mite-borne bacterium that causes the life-threatening human disease scrub typhus [2]. Small rodents serve as animal reservoirs for *O*. *tsutsugamushi*, but the bacterium can also be maintained within mite colonies through transovarial transmission. Symptoms in patients typically begin 6 to 10 days after inoculation by larval stage mites (‘chiggers’) and include headache, fever, rash, stupor, myalgia, and regional lymphadenopathy. Without appropriate treatment, the disease can progress to cause multiple organ failure and death. *O*. *tsutsugamushi* infections can be treated rapidly and effectively with tetracyclines, but the organism is intrinsically resistant to many common classes of antibiotics, including ß-lactams, fluoroquinolones, and aminoglycosides. Because of the generic nature of the symptoms, scrub typhus is difficult to diagnose unambiguously, and morbidity and mortality from scrub typhus typically result from delayed or ineffective treatment because of incorrect diagnosis. Natural immunity to scrub typhus is poor, and there is currently no vaccine available. High levels of antigenic diversity in *O*. *tsutsugamushi*, resulting from frequent genetic recombination as well as an antigenically variable major surface protein (TSA56), have posed a particular challenge to vaccine development. The diagnostic challenges associated with scrub typhus mean that its burden on public health has been previously underappreciated. However, numerous clinical studies have found it to be a leading cause of acute undifferentiated fever in regions of Southeast Asia, China, and India (e.g., [3]). Traditionally endemic in the Asia-Pacific, reports of locally acquired scrub typhus cases in Latin America and the Middle East [4,5] as well as the detection of rodents infected with species closely related to *O*. *tsutsugamushi* in Africa and Southern Europe [6,7] indicate that the disease may be more globally distributed.

### The genome of *O*. *tsutsugamushi*

The 2.1-megabase (Mb) single-chromosome genome of *O*. *tsutsugamushi* is the most highly repeated bacterial genome sequenced to date [8,9]. Around 42% of its genome is composed of repeated DNA, which includes short repetitive sequences, transposable elements (including insertion sequence elements, miniature inverted-repeat transposable elements, and a Group II intron), and a massively amplified integrative and conjugative element (ICE) called the rickettsial-amplified genetic element (RAGE). The RAGE, which has also been found in some other rickettsial genomes, is present in multiple partially degraded copies across the genome. This ICE contains integrase and transposase genes, *tra* genes typical of Type IV secretion systems, and some potential effector proteins, including ankyrin repeat–containing proteins, histidine kinases, and tetratricopeptide repeat (TPR) domain–containing proteins. Ankyrin repeat–containing proteins have been shown to be transcriptionally expressed in *O*. *tsutsugamushi*, secreted via a Type I secretion system, and localised to various host cell compartments, including the cytoplasm, nucleus, Golgi apparatus, and endoplasmic reticulum [10, 11]. Whilst some of the 359 Type IV secretion system *tra* genes have been shown to be expressed [12, 13], it is not known whether these form functional secretion systems nor what they might transport. Despite the genetic isolation of *O*. *tsutsugamushi* from other bacterial species resulting from its obligate intracellular lifestyle, the genome of *O*. *tsutsugamushi* exhibits a high degree of homologous recombination [14], and this may be mediated between *O*. *tsutsugamushi* strains through Type IV secretion systems. There is also evidence of some horizontal gene transfer from other bacterial species, including *Legionella* and *Parachlamydia* spp. [15]. As a consequence of the presence of multiple repeats and mobile DNA elements, the genome of *O*. *tsutsugamushi* has undergone extensive reshuffling, and there is very little correspondence between the positions of genes on the two complete published genomes of *Orientia* [8, 9] nor between *O*. *tsutsugamushi* and other closely related rickettsial genomes [16].

### Microtubule-mediated intracellular trafficking

**Fig 1:**
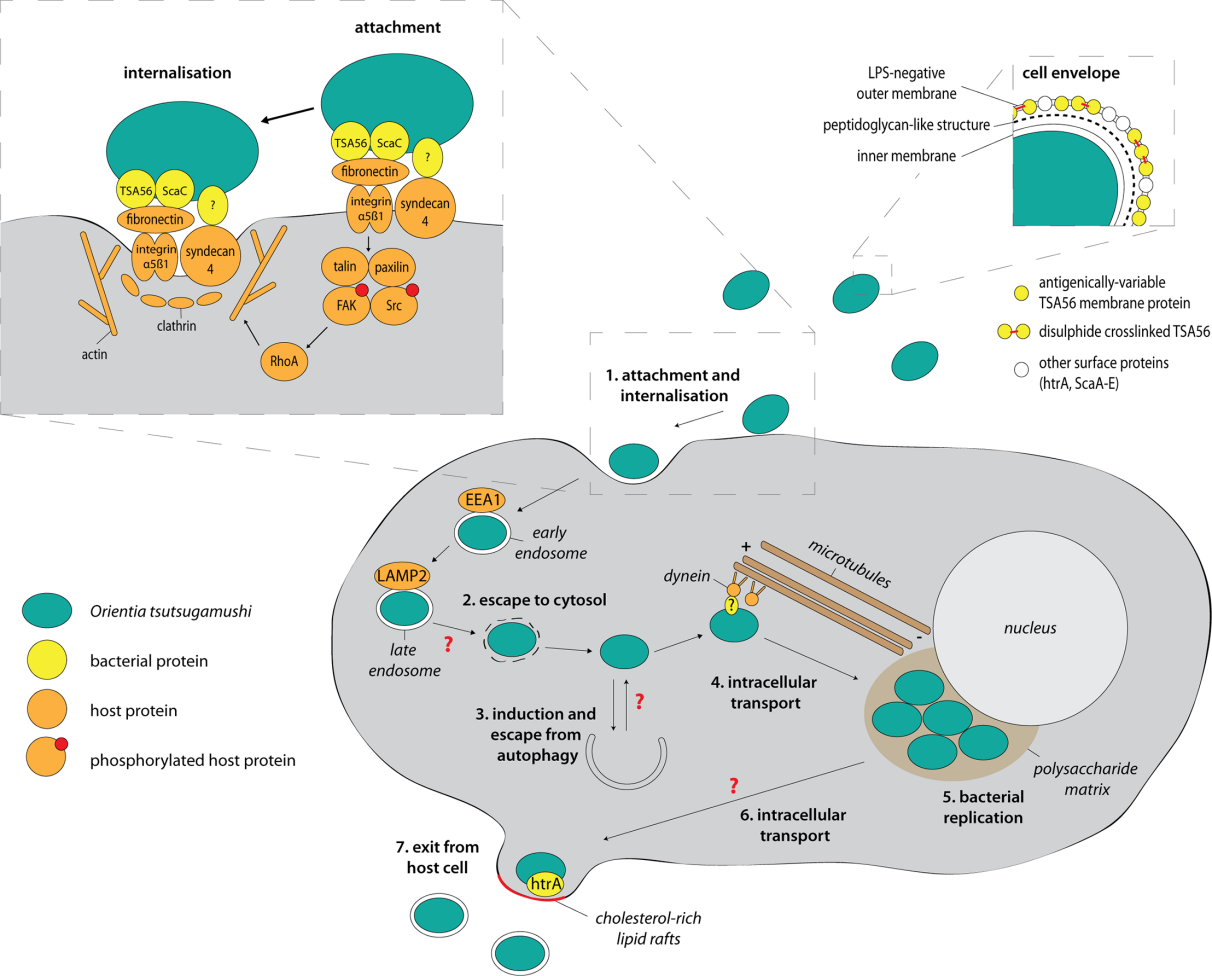
The cellular infection cycle of *O*. *tsutsugamushi*. Large inset shows a detailed view of the attachment and internalisation process. Small inset shows a schematic view of the cell envelope of *O*. *tsutsugamushi*. Red arrows indicate open questions and unknown pathways. LPS, lipopolysaccharide; ScaC, surface cell antigen C; TSA56, type surface antigen 56.

*O*. *tsutsugamushi* enters host cells through a clathrin-mediated zipper-like mechanism and escapes the endolysosomal pathway by exiting from late endosomes (Fig 1; reviewed in [17]). Once free in the cytoplasm, *O*. *tsutsugamushi* induces and evades autophagy [18, 19], then traffics to the perinuclear region, where bacterial replication takes place within a polysaccharide-enriched microcolony [20]. Whilst some bacterial species, including spotted fever–group rickettsias, *Shigella* and *Listeria*, employ actin-mediated processes to move through the viscous host cytoplasm [21], *O*. *tsutsugamushi* is unusual in employing microtubule-mediated processes for intracellular trafficking [22]—although some viruses such as adenoviruses and herpes simplex viruses as well as the bacterium *Aggregatibacter actinomycetemcomitans* also exploit this pathway [23]. The microtubule-mediated motility is dependent on the presence of polymerised microtubules as well as the minus-end–directed motor protein dynein [22]. It remains unknown which surface proteins on *O*. *tsutsugamushi* mediate the coupling to microtubule-binding proteins, how this is regulated, and why the perinuclear region is used for bacterial replication.

### Budding out of host cells

Intracellular pathogens typically exit infected host cells by host cell lysis (e.g., *Chlamydia* spp., *Plasmodium falciparum*), extrusion or expulsion of a bacteria-containing vacuole (e.g., *Chlamydia* spp., *Cryptococcus neoformans*), or actin-mediated protrusion into adjacent cells (e.g., *Listeria monocytogenes*, *S*. *flexneri*, *R*. *rickettsii*). *O*. *tsutsugamushi* employs an unusual budding mechanism to exit host cells, during which the exiting bacterium becomes encased in host plasma membrane (Fig 1) [24]. This process, which was shown to be dependent on cholesterol-rich lipid rafts as well as the bacterial surface protein HtrA [25], is highly reminiscent of that used by enveloped viruses. The formation of membrane-enclosed extracellular bacteria has implications for the mechanism of subsequent infection into naïve host cells, as well as providing a possible strategy for remaining hidden from the host immune system. Many important questions remain, such as the following: how does the bacterial cell bud without compromising the integrity of the remaining host plasma membrane? How stable is the plasma membrane around the budded bacterium? And is the encasing plasma membrane enriched for any specific membrane proteins or phospholipids?

### A minimal peptidoglycan-like structure in *O*. *tsutsugamushi*

*O*. *tsutsugamushi* was always reported to lack any peptidoglycan or lipopolysaccharide (LPS), but recent work has shown that the bacterium expresses peptidoglycan biosynthesis genes, is sensitive to cell wall–targeting drugs, and possesses a peptidoglycan-like structure [13, 26]. However, this structure is present at a low level and was difficult to detect. The shape of *O*. *tsutsugamushi* is less uniform than other Rickettsiaceae cells and most other peptidoglycan-positive rod-shaped bacteria, and this is consistent with the absence of a rigid cell wall. It is likely that *O*. *tsutsugamushi* has reduced the abundance of peptidoglycan in its cell wall because of its constant close proximity to cytosolic immune receptors such as nucleotide oligomerisation domain (NOD) proteins NOD1/NOD2 and that this was possible because of the osmotic protection of the cytosolic environment. The presence of a low-abundance, minimal peptidoglycan in *O*. *tsutsugamushi* is reminiscent of the cell wall of an unrelated group of obligate intracellular bacteria, the Chlamydiae. A comparison of the peptidoglycan-biosynthetic gene sets across these organisms reveals marked similarities, suggesting a conserved gene set required for building a minimal peptidoglycan structure. Both *Orientia* and *Chlamydiae* possess *murA-G* genes; *ftsW* and *rodA* from the shape, elongation, division, and sporulation (SEDS) protein family; and class B penicillin-binding proteins (PBPs), which have peptidoglycan transpeptidase activity. However, both notably lack any class A bifunctional PBPs that have both transpeptidase and glycosyltransferase activity.

### Concluding remarks

*O*. *tsutsugamushi* is an important pathogen that causes a high burden of severe disease in human populations. Several aspects of the biology of this obligate intracellular bacterium are particularly unusual, and these include a highly repeated genome abundant in mobile genetic elements, a dependence on microtubule trafficking for intracellular motility, a virus-like budding from infected host cells, and a *Chlamydia*-like minimal peptidoglycan cell wall. A more detailed understanding of these processes will yield a greater understanding of both general and unique aspects of the host–pathogen interface and illustrate the value of studying nonclassical model organisms.
